# Irreversible fatal contrast-induced encephalopathy: a case report

**DOI:** 10.1186/s12883-019-1279-5

**Published:** 2019-03-28

**Authors:** Wei Zhao, Jinping Zhang, Yun Song, Lili Sun, Meimei Zheng, Hao Yin, Jun Zhang, Wei Wang, Ju Han

**Affiliations:** Department of Neurology, Shandong Provincial Qianfoshan Hospital, Shandong University, Jinan, 250014 China

**Keywords:** Digital subtraction angiography, Iodinated contrast agents, Complications, Contrast-induced encephalopathy

## Abstract

**Background:**

Contrast-induced encephalopathy (CIE) is a well-known complication of iodinated contrast agents during angiography and vascular interventions. It can manifest as hemiparesis, cortical blindness, speech changes, Parkinsonism, confusion, seizure, and coma. Most of the reported CIE cases have been transient and reversible. Irreversible fatal CIE cases have been rarely reported. All the fatal CIE cases reported involved the use of ionic high osmolar contrast agents. Here, we document a heretofore unreported fatal CIE after digital subtraction angiography (DSA) using iopamidol, which is a type of non-ionic monomer low osmolar contrast agent.

**Case presentation:**

A 71-year-old woman was admitted to our Department of Neurology for tinnitus in the head. The cerebral magnetic resonance angiography (MRA) detected atherosclerotic cerebral arteries and bilateral stenosis of the middle cerebral arteries. The patient underwent DSA for further diagnostic work-up. The total amount of iopamidol used during the procedure was 110 ml. The patient experienced headache during the procedure, followed by dizziness with nausea and vomiting. Despite treatment with anti-oedema medications, her clinical status was gradually deteriorating and ended up with deep coma due to irreversible cerebral oedema which was confirmed by cerebral computed tomography (CT). Finally, the patient died 56 days after the procedure due to irreversible fatal cerebral oedema.

**Conclusions:**

This report documents that iopamidol-induced encephalopathy may not always have a benign outcome and can result in irreversible fatal cerebral oedema.

## Background

Contrast-induced encephalopathy (CIE) is a known but rare complication of angiography and endovascular interventions. The presentations may include hemiparesis, cortical blindness, speech changes, Parkinsonism, confusion, seizure, and coma [1, 2]. In most reported cases, the symptoms are reversible, and fatal encephalopathy following iodinated contrast administration has been rarely reported. Only 8 cases of autopsy-proven fatal cerebral oedema due to contrast neurotoxicity in the early stage of angiography have been reported [1, 3, 4]. All these reported fatal cases involved the use of high osmolar contrast agents. Iopamidol is a non-ionic monomer low osmolar contrast agent, which has been reported in cases of reversible contrast-induced encephalopathy [5–9]. Here, we describe a patient who suffered irreversible fatal encephalopathy after DSA using iopamidol.

## Case presentation

**Fig. 1:**
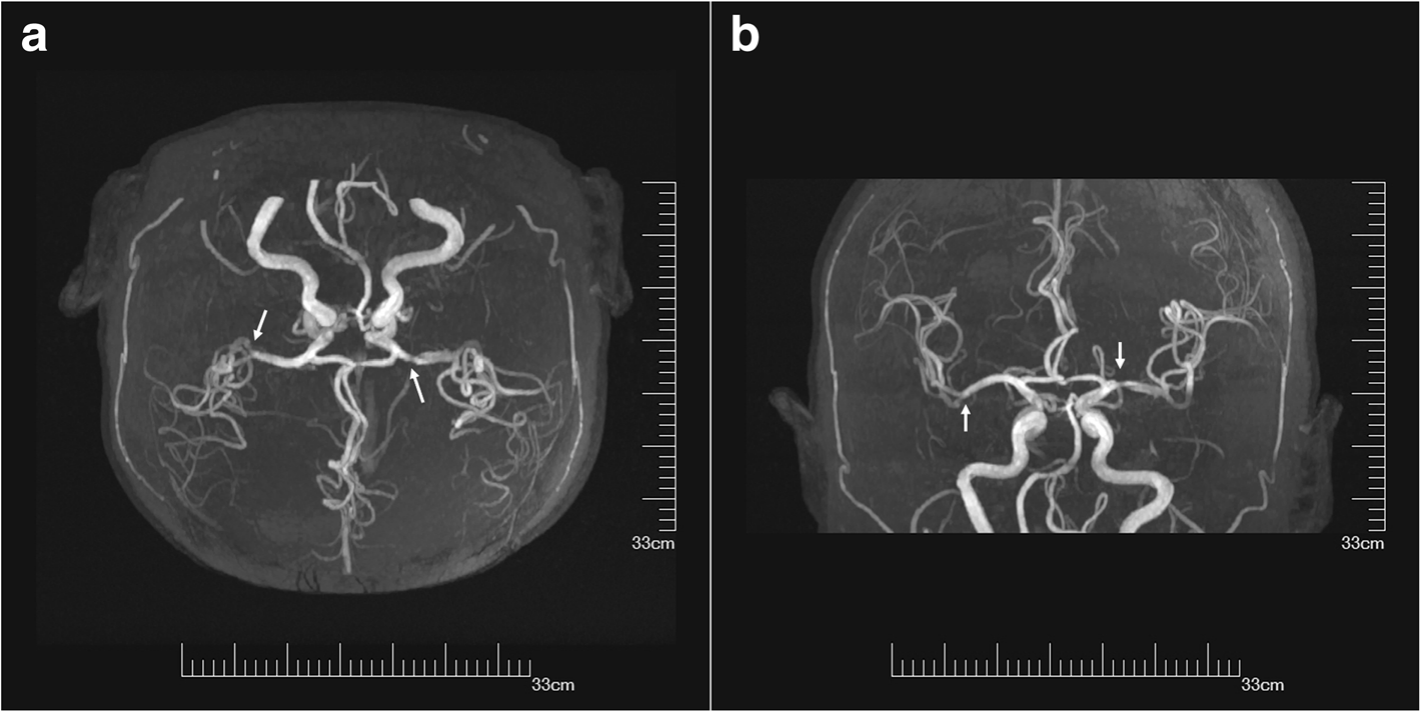
The cerebral MRA detected atherosclerotic cerebral arteries and bilateral stenosis of the middle cerebral arteries (**a**, **b** arrowheads), the stenosis of the left middle cerebral artery was serious

**Fig. 2:**
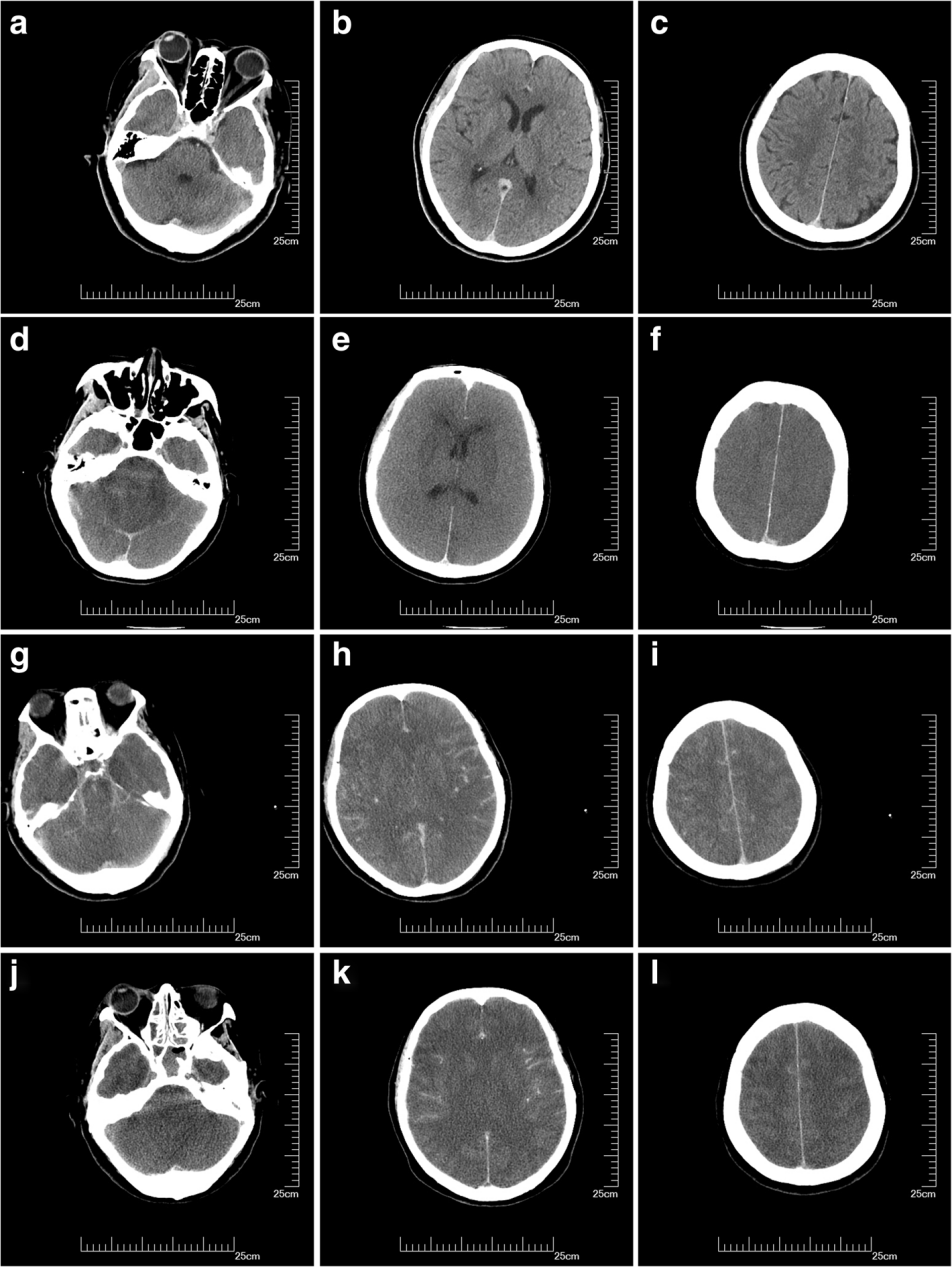
Cerebral CT immediately after DSA did not indicate any obvious abnormal sign (**a**, **b**, **c**); 2 days after the procedure, a repeat cerebral CT revealed diffuse cerebral oedema, loss of grey-white differentiation, effacement of the cerebral sulci and decrease in cerebrospinal fluid space (**d**, **e**, **f**); 9 days after the procedure, the cerebral CT showed more severe diffuse oedema of the brain, loss of grey-white differentiation, effacement of the cerebral sulci and subarachnoid space, disappearance of the cerebral ventricles, enhancement of the subarachnoid space, and darkened brain tissue in Hounsfield units (**g**, **h**, **i**); 15 days after the procedure, the cerebral CT showed persistent diffuse oedema of the brain with effacement of the cerebral ventricles and sulci, darkened brain tissue in Hounsfield units, and enhancement of the subarachnoid space (**j**, **k**, **l**)

A 71-year-old woman with a history of hypertension, hyperlipemia, and angina was admitted to our Department of Neurology for tinnitus in the head. On physical examination, bilateral hearing impairment was found. The cerebral magnetic resonance imaging (MRI) detected signal changes consisted with multiple cerebral infarctions and bilateral demyelination in the centrum semiovale. And the cerebral MRA detected atherosclerotic cerebral arteries and bilateral stenosis of the middle cerebral arteries (Fig. 1a, b). For further diagnosis, the patient underwent DSA subsequently. The total amount of iopamidol (Bracco Imaging Italia S.r.L.) administered during the procedure was 110 ml. The DSA showed that the patient had bilateral embryonic posterior cerebral arteries, 40% stenosis of the left middle cerebral artery and tortuous vertebral arteries bilaterally. There was no obvious calcification of the aortic arch; angiography of the arch using 25 ml iopamidol was performed only once. Ten minutes after the aortic arch angiography, the patient experienced mild headache. The pain was bearable, and the patient could cooperate during the remainder of the procedure. The DSA was completed 20 min later. No haemorrhage or vasospasm was detected during the procedure. The headache was continuous, and the patient suffered nausea and vomiting. The immediate physical examination showed no obvious abnormal sign. The patient was treated with 8 mg ramosetron and 10 mg dexamethasone. After 20 min of observation, the symptoms were relieved. Her cerebral CT scan at the time was normal (Fig. 2a, b, c). Two hours later, the patient manifested dizziness with nausea and vomiting and was treated with 8 mg ondansetron and 20 mg diphenhydramine. Meanwhile, compound sodium chloride injection was used to facilitate the elimination of the contrast agent. The treatment alleviated her symptoms. Four hours after the procedure, the patient re-experienced dizziness; thus 5 mg dexamethasone was administered but resulted in no alleviation until after 11 h wherein dizziness was relived but her blood pressure was 183/92 mmHg. The patient was drowsy but could answer questions correctly. The pupil were symmetric and reactive, and limb movements were preserved. To manage hypertension, 30 mg nimodipine tablets were used. Fourteen hours after the procedure, the patient fell asleep, but at 17 h, respiratory failure progressed and oxygen saturation dropped to 88%. The patient was in a coma state, with sighing respiration. Anisocoria with non-reactive pupils developed, limb drop test was positive along with flexor plantar and Babinski sign was negative. Therefore, cerebral hernia was considered. The patient was treated with 20% mannitol, nikethamide, lobeline and diprophylline, and was transferred to the intensive care unit for further treatment after cardio-pulmonary resuscitation, endotracheal intubation and mechanical ventilation. Two days after the procedure, cerebral CT scan indicated diffuse cerebral oedema, loss of grey-white differentiation, effacement of the cerebral sulci and decrease in cerebrospinal fluid space (Fig. 2d, e, f). The patient was treated with dehydration, mechanical ventilation, and anti-infectious agent, but the diffuse cerebral oedema did not improve. Nine days after the procedure, the third cerebral CT scan showed that the cerebral oedema had become much more severe, the ventricles had disappeared and there was hyperdense signal in the subarachnoid space, which was considered to be indicative of a pseudo-subarachnoid haemorrhage due to the severe cerebral oedema [10] (Fig. 2g, h, i). Fifteen days after the procedure, the cerebral CT scan detected unrelieved diffuse cerebral oedema, and the hyperdense signal in the subarachnoid space persisted (Fig. 2j, k, l). None of these cerebral CT scans showed intracerebral haemorrhage or infarct in this patient. The patient remained in a continuous deep coma state, and the brainstem reflexes had disappeared; she died 56 days after that sudden deterioration.

## Discussion and conclusions

The prognosis of most CIE is generally reported to be good with a rapid recovery, and only rare cases with the persistent deficits have been reported [2]. Notably, there were 8 cases of autopsy-proven fatal cerebral oedema due to contrast neurotoxicity in the early stage of angiography [1, 3, 4]. The 8 deaths included 6 infants; 5 of these patients underwent cardiac angiography, and the other 3 received aortography. All fatal cerebral oedema cases reported involved the use of ionic high osmolar contrast agents, and ionic high osmolar contrast agents are no longer used in routine angiography and intervention procedures. The case which we report here may be the first fatal cerebral oedema after DSA using iopamidol. This case highlights the potential for other types of iodinated contrast agents to induce fatal encephalopathy.

The diagnosis of CIE is important, as its presentation may be similar to embolism, and haemorrhagic complications following angiography or endovascular interventions. Typical radiological findings include abnormal cortical contrast enhancement and cerebral oedema, subarachnoid contrast enhancement and striatal contrast enhancement [2, 11]. CT or MRI of the brain helps us to differentiate CIE from haemorrhage or infarct. In the case which we report here, none of the CT scans of the brain after DSA (immediately, 2 days, 9 days and 15 days after the procedure) indicated intracerebral haemorrhage or infarct. Therefore, the possibility of multiple embolisms was not considered in this case. The hyperdense signal in the subarachnoid space in the cerebral CT scans was considered to be due to the severe diffuse cerebral oedema. The hyperdense appearance results from a combination of loss of grey-white differentiation, narrowing and effacement of the subarachnoid spaces, and corresponding engorgement of superficial pial veins [10].

The patient experienced headache 10 minutes after the aortic arch angiography during the DSA procedure, and suffered nausea, vomiting and dizziness after the procedure. The symptoms were continuous, the patient was comatose at 17 h with respiratory failure. Anisocoria with non-reactive pupils developed, the limb drop test was positive, and Babinski sign was negative. All these symptoms supported the diffuse lesion of the brain, there was no sign of focal brain lesion. The cerebral CT scans detected diffuse unrelieved cerebral oedema after the severe deterioration. Therefore, cerebral hernia was considered due to severe cerebral oedema. The sudden respiratory failure was due to cerebral oedema leading to cerebral hernia. Hence the anoxic brain injury was not considered in this case. And the history, symptoms and cerebral CT scans did not support the diagnoses of secondary viral encephalitis, secondary cerebral vein thrombosis and metabolic causes.

The mechanism of CIE is controversial. The temporary disruption of the blood-brain barrier (BBB) after injection of the iodinated contrast agent is widely accepted [2, 11–15]. Experimental studies have demonstrated that contrast agents can penetrate the altered BBB and that this is dependent on the contact time, anions and dosage [1, 12, 13, 15]. Both the hyperosmolality and chemotoxicity of the contrast agents contribute to the disruption of the BBB. Hyperosmolality of the contrast medium is hypothesised to cause shrinkage of endothelial cells and disrupt tight junctions [12]. Other studies suggest that the alteration of the BBB is due to the physical or chemical effects of the contrast medium on the BBB instead of the hyperosmolality [14]. The expression of endothelin, which can be induced by radiocontrast agents, can increase human brain endothelial cell permeability and has been implicated in the pathophysiology of disorders associated with BBB injury [2, 15].

Studies have indicated that opening of the BBB may be accompanied by brain oedema, resulting from the flux of proteins, electrolytes, and water across the abnormally permeable cerebral vessels into the extracellular space [4]. An idiosyncratic response to small doses of contrast agent, which may be related to the areas of incompleteness of their BBB, has been reported [1]. We postulate that the idiosyncratic response to contrast agents may have contributed to the patient’s prolonged and progressive brain oedema. Contrast agents can produce direct neurotoxic effects on the neurons and astrocytes when they penetrate the altered BBB. Experimental studies have shown that ionised contrast agents can severely alter neuronal function when directly introduced into the nervous system [1, 12, 13, 15]. We hypothesised that the direct neurotoxic effect of the contrast agent also contributed to the patient’s progressive and fatal brain oedema.

All types of iodinated contrast agents can induce the development of neurotoxicity, but the occurrence of fatal cerebral oedema is very rare. Unfortunately, there is no currently available effective treatment for such a severe fatal CIE. In the case reported by L. Junck and W.H. Marshall [4], the post mortem tissue iodine concentrations were the highest in the urine, serum and kidney. The use of continuous renal replacement therapy and continuous blood purification may be potential treatments for cases of fatal CIE.

In summary, although CIE has typically been associated with benign outcomes in previous studies, we present a case of fatal cerebral oedema after DSA using iopamidol. This case illustrates the potential to cause severe complications, even fatal cerebral oedema, with all types of iodinated contrast agents. The doctors performing angiography and interventions should be aware of this severe potentially harmful effect. The rare occurrence of severe contrast-induced complications renders their prevention very difficult. Further studies are needed to define the risk factors and the mechanism of the iodinated contrast agent neurotoxicity, which may help minimise the occurrence of severe complications.

## Abbreviations

BBB: Blood-brain barrier
CIE: Contrast-induced encephalopathy
CT: Computed tomography
DSA: Digital subtraction angiography
MRA: Magnetic resonance angiography
MRI: Magnetic resonance imaging

## References

[1] Lalli AF (1980). Contrast media reactions: data analysis and hypothesis. Radiology.

[2] Leong S, Fanning NF (2012). Persistent neurological deficit from iodinated contrast encephalopathy following intracranial aneurysm coiling. A case report and review of the literature. Interv Neuroradiol.

[3] Shrivastava S, Mohan JC, Chopra P (1985). Fatal cerebral edema following angiocardiography: a case report. Int J Cardiol.

[4] Junck L, Marshall WH (1986). Fatal brain edema after contrast-agent overdose. AJNR Am J Neuroradiol.

[5] Parry R, Rees JR, Wilde P (1993). Transient cortical blindness after coronary angiography. Br Heart J.

[6] Kamata J, Fukami K, Yoshida H, Mizunuma Y, Moriai N, Takino T (1995). Transient cortical blindness following bypass graft angiography. A case report. Angiology.

[7] Uchiyama Y, Abe T, Hirohata M, Tanaka N, Kojima K, Nishimura H (2004). Blood brain-barrier disruption of nonionic iodinated contrast medium following coil embolization of a ruptured intracerebral aneurysm. AJNR Am J Neuroradiol.

[8] Gellen B, Remp T, Mayer T, Milz P, Franz WM (2003). Cortical blindness: a rare but dramatic complication following coronary angiography. Cardiology.

[9] Merchut MP, Richie B (2002). Transient visuospatial disorder from angiographic contrast. Arch Neurol.

[10] Hasan TF, Duarte W, Akinduro OO, Goldstein ED, Hurst R, Haranhalli N (2018). Nonaneurysmal "pseudo-subarachnoid hemorrhage" computed tomography patterns: challenges in an acute decision-making heuristics. J Stroke Cerebrovasc Dis.

[11] Lantos G (1989). Cortical blindness due to osmotic disruption of the blood-brain barrier by angiographic contrast material: CT and MRI studies. Neurology.

[12] Junck L, Marshall WH (1983). Neurotoxicity of radiological contrast agents. Ann Neurol.

[13] Torvik A, Walday P (1995). Neurotoxicity of water-soluble contrast media. Acta Radiol Suppl.

[14] Wilson AJ, Evill CA, Sage MR (1991). Effects of nonionic contrast media on the blood-brain barrier. Osmolality versus chemotoxicity. Investig Radiol.

[15] Heyman SN, Clark BA, Kaiser N, Spokes K, Rosen S, Brezis M (1992). Radiocontrast agents induce endothelin release in vivo and in vitro. J Am Soc Nephrol.

